# Effects of Cord Blood Serum (CBS) on viability of retinal Müller glial cells under in vitro injury

**DOI:** 10.1371/journal.pone.0234145

**Published:** 2020-06-04

**Authors:** Carmen Ciavarella, Marina Buzzi, Elisa Bergantin, Stefano Di Marco, Giuseppe Giannaccare, Emilio Campos, Silvia Bisti, Piera Versura

**Affiliations:** 1 Ophthalmology Unit, DIMES, Alma Mater Studiorum University of Bologna, S.Orsola-Malpighi Teaching Hospital, Bologna, Italy; 2 Emilia Romagna Cord Blood Bank-Transfusion Service, S.Orsola-Malpighi Teaching Hospital, Bologna, Italy; 3 Vision Lab, DISCAB, University of L’Aquila, L’Aquila, Italy; 4 Istituto Italiano di Tecnologia (IIT), Genova, Italy; National Institutes of Health, UNITED STATES

## Abstract

Oxidative stress and inflammation determine retinal ganglion cell degeneration, leading to retinal impairment and vision loss. Müller glial cells regulate retinal repair under injury, through gliosis. Meanwhile, reactive gliosis can turn in pathological effects, contributing to neurodegeneration. In the present study, we tested whether Cord Blood Serum (CBS), rich of growth factors, might improve the viability of Müller cells under in vitro damage. BDNF, NGF, TGF-α, GDNF and EGF levels were measured in CBS samples by Human Magnetic Luminex Assay. CBS effects were evaluated on rat (rMC-1) and human (MIO-M1) Müller cells, under H_2_O_2_ and IL-1β damage. Cells grown with FBS or CBS both at 5% were exposed to stress and analyzed in terms of cell viability, GFAP, IL-6 and TNF-α expression. CBS was also administrated after treatment with K252a, inhibitor of the neurotrophin receptor Trk. Cell viability of rMC-1 and MIO-M1 resulted significantly improved when pretreated with CBS and exposed to H_2_O_2_ and IL-1β, in comparison to the standard culture with FBS. Accordingly, the gliosis marker GFAP resulted down-regulated following CBS priming. In parallel, we observed a lower expression of the inflammatory mediators in rMC-1 (TNF-α) and MIO-M1 (IL-6, TNF- α), especially in presence of inflammatory damage. Trk inhibition through K252a administration impaired the effects of CBS under stress conditions on MIO-M1 and rMC-1 viability, not significantly different from FBS condition. CBS is enriched with neurotrophins and its administration to rMC-1 and MIO-M1 attenuates the cytotoxic effects of H_2_O_2_ and IL-1β. Moreover, the decrease of the main markers of gliosis and inflammation suggests a promising use of CBS for neuroprotection aims. This study is a preliminary basis that prompts future investigations to deeply explore and confirm the CBS potential.

## Introduction

Oxidative stress plays a pivotal role in the pathogenesis of many neurodegenerative diseases, including those affecting the retina [1,2]. The production of reactive oxygen species (ROS) stimulate the inflammatory response, by inducing the release of different inflammatory mediators, which contribute to cell death and retinal degeneration [3]. At this regard, several studies suggest that neuroinflammation is a core element in many retinal diseases for the progression of neurodegenerative processes, thus targeting the neuroinflammatory pathway has been recently proposed as a promising therapeutic strategy [4,5].

Müller cells represent the main type of retinal glial cells, spanning the whole thickness of the retina and performing a wide range of functions aimed at keeping the retinal homeostasis and health [6]. Müller cells orchestrate the retinal response to stress [6], but they can be considered a primary pathogenic mediator when their response is overdue [7]. Müller cells ensure the nourishment and the functioning of the retinal neurons, allowing their connection with blood vessels, vitreous body and subretinal spaces for substance exchange [8,9]. Together with retinal astrocytes, Müller cells preserve the inner blood-retinal-barrier (BRB), regulate the extracellular ion changes, pH and neurotransmitter homeostasis. Also, they possess antioxidant activity by which they reduce the oxidative stress. Under stress conditions, like ROS- and inflammation- induced injury, Müller cells undergo structural and functional changes, a process known as gliosis. Hypertrophy, activation of the extracellular signal-regulated kinases (ERK) and up-regulation of the intermediate filament proteins, glial fibrillary acidic protein (GFAP), vimentin and nestin, are the hallmark of reactive gliosis [10,11]. This process normally serves as a protective mechanism for retinal tissue from damage and is accompanied by the secretion of antioxidant molecules and neuroprotrophic factors, which promote neuron growth and survival. This group includes neurotrophins, like brain derive neurotrophic factor (BDNF) and nerve growth factor (NGF). However, the chronic activation of this circuit evolves into a pathological mechanism with detrimental effects, defined as massive or proliferative gliosis [12,13]. This condition exacerbates the retinal disease, and it has been observed in many neurodegenerative disorders, including glaucoma, age-related macular degeneration and diabetic retinopathy [13]. Considered their multifaceted nature and behavior, Müller cells represent a putative cell target for testing novel therapeutic strategies for retinal diseases.

Recently, it has been drawn attention to the neuroprotective effects of trophic factors in retinal diseases [14–17], and the possibility to deliver these factors by eye-drops [15,18,19]. The efficacy of different factors like BDNF, NGF, ciliary neurotrophic factor (CTNF), glial cell-derived neurotrophic factor (GDNF) might be different, depending on cell types and receptors that are differentially located and expressed in a variety of physiological and pathological conditions [5,14,20].

Cord blood (CB) is rich of trophic factors, including neurotrophins such as BDNF, NGF, GDNF, Transforming Growth Factor (TGF)-α, and Epidermal Growth factor (EGF) [21,22]. Some of them, obtained with recombinant technologies, have been already tested separately, with results still in progress in the field of retinal neuroprotection [23–27]. CB is the outcome of a period of high metabolic demand during pregnancy, and it might therefore represent the most powerful combination of natural trophic factors [22].

Based on these premises, the present study was aimed at investigating whether the supplementation of culture media with CBS can reduce the in vitro response of Müller cells under stress conditions.

## Materials and methods

### CBS collection and preparation

The study was performed in respect to the principles in the Declaration of Helsinki. The use of the CBS was approved by the local Ethical Committee for the use of human products for research (Comitato Etico Indipendente dell’AOU di Bologna, Policlinico S.Orsola-Malpighi, approval n. 128/2017/U/Sper).

CB units were collected at the time of delivery after compilation of a donor selection questionnaire and signing informed consent; all steps from the recruitment to the processing and registration of CB were performed according to standard operating procedures and guidelines issued by the Foundation for the Accreditation of Cellular Therapy (FACT). CB was collected from spontaneous term births free of complications (≥37^th^ week of pregnancy) and Caesarean births decided by trained and qualified health personnel. CB collection for research purpose was performed from ex utero placenta vessels, and transferred in 9 ml Vacumtube (Biomed Device, Italy) without any anticoagulant. The medium volume of collected samples was 7±1,5ml. For further processing, the samples were sent to the Processing Facility (PF) laboratory of the CB Bank opened 24/24hrs, 7/7 days, where it went through a series of checks and tests to establish the blood characteristics and its suitability for preservation and therapeutic use. Maternal infectious disease markers (HIV, HCV, HBV Treponema pallidum, CMV, Toxoplasmosis and HTLVI/II) evaluations were performed. Suitable samples were centrifuged at 2.800 g for 10 min and then transferred in sterile tubes under laminar flow hood and stored at -80°C. CB samples had an average volume of 4 ± 0.5 ml. CBS samples were pooled and one aliquot of this pool was tested for the presence of selected growth factors: BDNF (Brain Derived Neurotrophic Factor), NGF (Nerve Growth Factor), GDNF (Glial Derived Neurotrophic Factor), TGF (Transforming Growth Factor)-α, and EGF (Epidermal Growth factor). The analysis was repeated after heat treatment (56°C, 20 min) for complement inactivation.

### Cell culture and reagents

In this study human and rat retinal Müller cells were used. Immortalized rat Muller cells (rMC-1, T0576) were purchased from ABM Good (Richmond, BC, Canada) and grown in Prigrow medium 1% antibiotics. For the human model, a spontaneously immortalized human Muller cell line MIO-M1 (Moorfields/Institute of Ophthalmology-Muller 1) was obtained from the UCL Institute of Ophtalmology, London, UK [28]. According to the manufacture’s instructions, MIO-M1 were cultured in DMEM L-Glutamax (Gibco, Thermofisher Scientific). FBS or CBS were added to growth medium at 5%. Hydrogen peroxide (H_2_O_2_) at 30% (Sigma Aldrich) was used to induce oxidative stress and was diluted in sterilized water at 9.88 mM, before preparation of working solutions in serum-free medium for cell treatments. Interleukin-1β (Sigma Aldrich) was chosen for the inflammatory stimulation and was diluted in sterilized water at 10 μg/ml, before addition to serum-free cultures.

### Cell treatments and experimental design

The effects of oxidative stress and inflammation in presence of FBS or CBS were evaluated in terms of cell viability in rat and human Muller cells. The treatment scheme was adjusted depending on cell model reactivity and concentration/time points for H_2_O_2_ and IL-1β differ between rat and human.

### Cell viability assay

Cell viability was determined by MTT assay (Vybrant MTT Cell Proliferation Assay Kit, Life Technologies) following the manufacturer’s instructions. To this scope, cells were seeded in 96-well plates at 10^4^ cells/well in 100 μl growth medium containing 5% FBS or CBS. Cells were treated with H_2_O_2_ (100-200-500 μM) and IL-1β (10–50 ng/ml), in serum-free medium. The MTT assay was performed on rMC-1 after 3-6-24 h with H_2_O_2_ and 24 h with IL-1β, on MIO-M1 for 24 h for both treatments. Briefly, cell medium was replaced with fresh growth medium after treatment, and 10 μl of 12 mM MTT component A (3-(4,5-dimethylthiazol-2-yl)-2,5-diphenyltetrazolium bromide) were added to each well, and left in incubator for 4 h. Then, 100 μl of MTT component B sodium dodecyl sulfate (SDS)-hydrochloride acid (HCl) were added for further 18 h at 37°C. Absorbance was red at optical density (O.D.) 570 nm by microplate reader. Cell survival rate was normalized to rMC-1 and MIO-M1 grown in medium without serum, in order to exclude the decrease of cell viability due to the lack of serum.

### Tropomyosin receptor kinase (Trk) inhibition

For the inhibition of Trk receptor, a novel experimental set was designed. Briefly, cells were treated with 200 nm of K252a (K1639, Sigma Aldrich) for 1 h. Then, the culture medium was changed with FBS or CBS at 5% for 24 h, followed by addition of H_2_O_2_ (200 μM in MIO-M1 and 100 μM in rMC-1) and IL-1β (50 ng/ml) for further 24 h. MTT assay was performed, as above described, at the end of treatment for the analysis of cell viability.

### RNA extraction and cDNA reverse transcription

Total RNA extraction was performed by using TRIreagent (TRIzol reagent, Life Technologies) according to the manufacturer’s instructions. RNA concentration was evaluated by QUBIT RNA Broad Range (Life Technologies) and one μg of total RNA was reverse transcribed in a 20 μl reaction volume using High Capacity Reverse Transcription Kit (Life Technologies). Real Time PCR was carried out in a CFX Connect Real-Time PCR Detection System (BioRad), using TaqMan (TaqMan Master Mix, Life Technologies) and Sybr Green approach (Sybr Green Real Time PCR Master Mix, Life Technologies). Each assay was executed in triplicate and target gene expression was normalized to β-actin (rat) and glyceraldehyde 3-phosphate dehydrogenase (GAPDH) (human). The final results were determined by the comparative 2^^-ΔΔCt^ method and expressed as fold changes relative to untreated control cultured with growth medium without serum.

The probes used for TaqMan assays were purchased from Life Technologies and are listed in Table 1. The primer sequences for Sybr Green were designed by Primer Blast and purchased from Sigma Aldrich (Table 1).

**Table 1:** List of primer sequences/probes used for Real Time experiments.

Gene	Primer sequence/Probe accession number
Rat β-actin Probe	Rn00667869_m1
Rat GFAP Probe	Rn00566603_m1
Rat TNF-α Probe	Rn01525859_g1
Human GAPDH 5’Primer 3’Primer Probe	AATGGGCAGCCGTTAGGAAA AGGAGAAATCGGGCCAGCTA Hs02786624_g1
Human GFAP Probe	Hs00909233_m1
Human TNF-α	
5’Primer 3’Primer	CAGGGACCTCTCTCTAATCA TTGAGGGTTTGCTACAACAT
Human IL-6 5’Primer 3’Primer	TCAATATTAGAGTCTCAACCCCCA GAAGGCGTTGTGGAGAAGG

GFAP: glial fibrillary acidic protein; TNF-α: tumor necrosis factor α; IL-6: interleukin 6.

### Immunofluorescence

Immunofluorescence was assessed to detect the expression of GFAP and Trk receptor in MIO-M1. Briefly, 15000 cells were seeded on glass slides in growth medium enriched with FBS or CBS at 5%. After 24 hours, rMC-1 and MIO-M1 were exposed to H_2_O_2_ and IL-1β according to the experimental scheme. After each treatment, cells were washed with PBS and fixed/permeabilized with ice-cold methanol for 10 minutes at room temperature. Non-specific binding was allowed through 30 minute incubation with 1% bovine serum albumin (BSA) in PBS, at RT. Slides were then labeled with monoclonal antibodies in 1% BSA/PBS for 1 h at 37°C in a wet chamber. Antibody dilutions were the following: GFAP (1:500, Synaptic System), TrkA/B/C (1:50, Life Technologies). After PBS washes, the cells were stained with anti-mouse AlexaFluor-488 (1: 250, Life Technologies), or anti-rabbit AlexaFluor-546 (1: 300, Life Technologies), secondary antibody for 1 h at 37°C in the dark and counterstained with ProLong antifade reagent with DAPI (Life Technologies). Finally, the slides were observed in a Leica DMI6000 B inverted fluorescence microscope (Leica Micro-systems, Wetzlar, Germany). Quantification of Trk receptor expression was performed on digitalized images randomly acquired at 20X original magnification, with a minimum of 5 fields examined for each condition. Data were measured as fluorescence intensity by using the software ImageJ (http://rsb.info.nih.gov/ij) and reported as mean of positive areas normalized to total area.

### Statistical analysis

Each experiment was executed at least in triplicate. Results were analyzed by GraphPad Prism 6 statistical software (GraphPad Software Inc) and expressed as mean ± standard deviation. Statistical analysis was performed using unpaired t test, one-way and two-way ANOVA test followed by Tukey and Sidak multiple comparisons test. Results were considered statistically significant at the 95% confidence level (p value < 0.05 was considered as significant).

## Results

Experimental results have been organized in four subsequent steps: 1) optimization of the damage induction scheme in rat and human Müller cells; 2) preparation of CBS and dosage of neurotrophins; 3) in vitro testing of CBS on Müller cells under stress conditions; 4) inhibition of neurotrophin receptor before CBS administration in rat and human Müller cells.

### Oxidative stress and inflammatory damage on Müller cells

**Fig 1:**
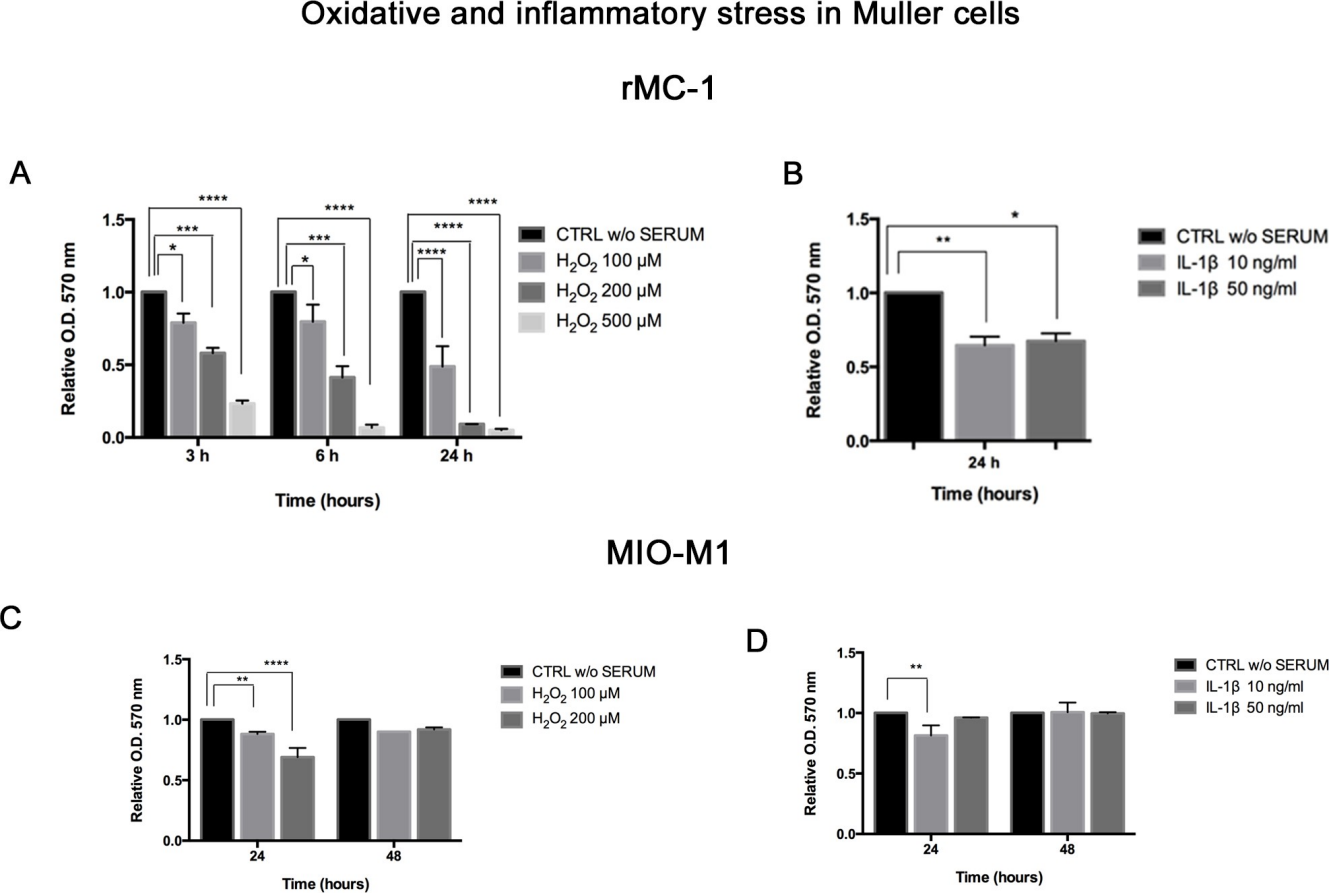
Effects of oxidative stress and inflammation on Müller glial cells. H_2_O_2_ and IL-1β were administrated to rat (rMC-1) and human (MIO-M1) Müller cells to mimic the neurodegenerative damage. Times and concentrations were different between the two cell lines, according to previous experimental setting. Viability of: rMC-1 with (A) oxidative stress for 3-6-24 h and (B) inflammatory stimulation for 24 h; MIO-M1 with (C) oxidative stress and (D) inflammatory stimulation for 24–48 h. Results were normalized to untreated controls, in DMEM without serum (CTRL w/o SERUM), as relative absorbance measured at an optical density (O.D.) of 570 nm, are reported as mean ± standard deviation of three independent experiments. *, p<0.05; **, p<0.01; ***, p<0.001; ****, p<0.0001 (Ordinary one- and two-way Anova, followed by Tukey’s multiple comparison tests).

Oxidative stress and inflammation are central to most of the ocular neurodegenerative pathways, stimulating Müller cell activation to repair the damage. In this study, rMC-1 and MIO-M1 were exposed to increasing concentrations of H_2_O_2_, as inducer of oxidative stress, and IL-1β for the activation of the inflammatory cascade. Both treatments were administrated in growth medium without serum. A preliminary viability assay was performed to set the experimental doses and times, and to exclude the sub lethal treatments, which resulted different for each cell line. rMC-1 were treated with H_2_O_2_ ranging from 100 to 500 μM for 3–6 and 24 hours. Cell viability, evaluated through MTT assay, resulted decreased in a dose- and time-dependent manner. The most detrimental effects were recorded after 24 h exposure, where a 95% and 97% decrease of cell viability was observed in presence of 200 μM and 500 μM of H_2_O_2_, respectively (Fig 1A). Treatment for 3 and 6 hours were less cytotoxic; however, H_2_O_2_ at 500 μM was confirmed as the most harmful concentration, recording the 80% and 90% of cell death at 3 and 6 h, respectively (Fig 1A). Thus, 500 μM H_2_O_2_ and treatment for 24 h were excluded from this experimental design. IL-1β administration for 24 h exerted a significant effect on rMC-1, determining a 40% lowering of cell viability (Fig 1B).

MIO-M1 viability was measured at longer exposures (24–48 h), since short time treatments did not exert effects comparable to those observed in rMC-1. We observed a reduction of cell viability of almost 20% and 30% in presence of 100 μM and 200 μM of H_2_O_2,_ respectively, for 24 h (Fig 1C). A 20% decrease of MIO-M1 survival was also reported under inflammatory conditions, with both IL-1β concentrations at 24 h (Fig 1D).

### Neurotrophin levels in CBS

Human CBS samples were prepared for administration to cell cultures. One aliquot of each CBS sample was pooled destined to neurotrophin dosage by Human Magnetic Luminex Screening Assay, preliminarily to the usage for cell cultures. Single samples of CBS were pooled and the resulting median values of measured growth factors in the resulting pool are reported in Table 2. The levels of neurotrophins BDNF and NGF were analyzed before and after heat inactivation (in a lot not utilized for the experiments due to a low neurotrophin levels), with no significant changes of growth factor concentrations (S1 Fig).

**Table 2:** Growth factors levels in human CBS samples.

Growth factors	Concentrations
BDNF	15.6 ng/ml
NGF	3.0 pg/ml
GDNF	1.5 pg/ml
TGF-α	36.3 pg/ml
EGF	820 pg/ml

BDNF: Brain Derived Neurotrophic Factor; NGF: Nerve Growth Factor; GDNF: Glial Cell-Derived Neurotrophic Factor; TGF-α: Transforming Growth Factor alpha; EGF: Epidermal Growth Factor.

These data were further compared with the batch of FBS used in the present study, and the concentration levels of the growth factors resulted lower than CBS (BDNF: 330 ± 50 pg/ml; the other growth factors were under the threshold of detection).

### CBS reduces the mortality rate of rMC-1 exposed to H_2_O_2_

**Fig 2:**
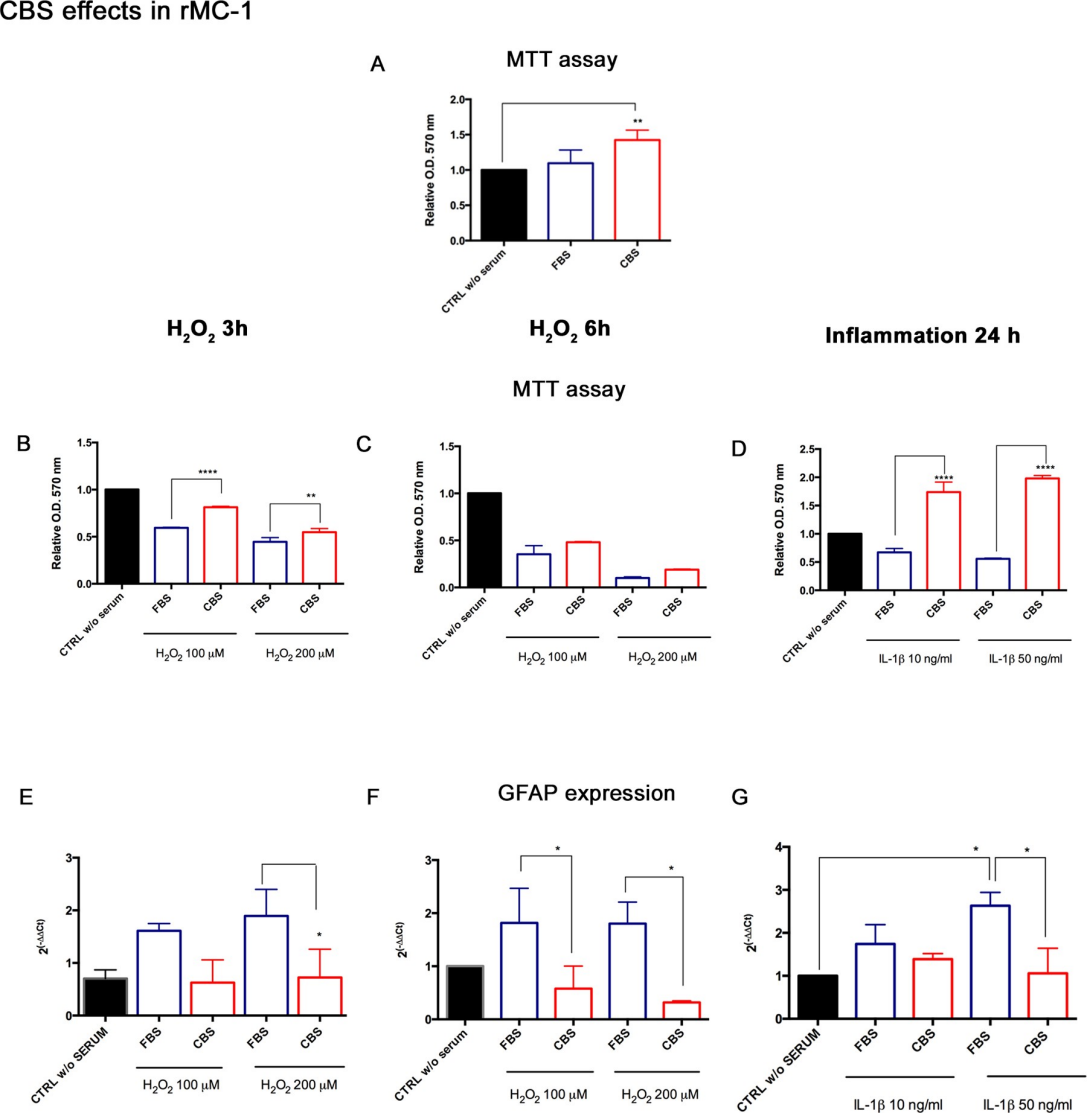
CBS ameliorates rMC-1 viability and keep low GFAP expression under stress conditions. (A) The effects of CBS on normal rMC-1 viability were preliminarily evaluated after 24 h e measured by MTT assay. Then, rMC-1 were grown with FBS or CBS, and then were exposed to oxidative and inflammatory stress. Cell viability was measured by MTT assay after (B) 3 and (C) 6 hours with 100 and 200 μM H_2_O_2_, and (C) 24 h after exposure to IL-1β (10–50 ng/ml). The GFAP gene was analyzed by Real Time under oxidative stress (D, E) and inflammation (F). MTT data were normalized to untreated controls, in DMEM without serum (CTRL w/o SERUM), as relative absorbance measured at an optical density (O.D.) of 570 nm, are reported as mean ± standard deviation of three independent experiments. Real Time PCR are reported as fold changes relative to untreated controls, in DMEM without serum, and are expressed as mean ± standard deviation of three independent experiments. Statistical analysis was performed by ordinary one-way Anova, followed by Tukey’s multiple comparisons test. *, p<0.05; ****, p<0.0001.

CBS was tested in Müller cells under stress conditions, mimicked both by oxidative stress and inflammation. We preliminarily analyzed whether CBS administration affects the rMC-1 viability, and we did not observe significant variation in rMC-1 growth between FBS and CBS (Fig 2A). Thus, rMC-1 cultured with CBS or FBS processed for viability assessment after 3- and 6-hour treatment with H_2_O_2_. CBS improved the cell survival under oxidative stress, and the MTT assay revealed significant differences between CBS and FBS, especially after short-term exposure (100 μM H_2_O_2_ for 3 h) (Fig 2B and 2C). Significant differences in cell viability were observed under inflammatory injury. rMC-1 grown with CBS exhibited higher MTT activity than FBS, with both inflammatory cytokine concentration (Fig 2D).

### CBS reduces GFAP and TNF-α in rMC-1

GFAP and TNF-α were analyzed in rMC-1, in order to evaluate whether CBS could decrease the gliosis activation and the inflammatory state.

**Fig 3:**
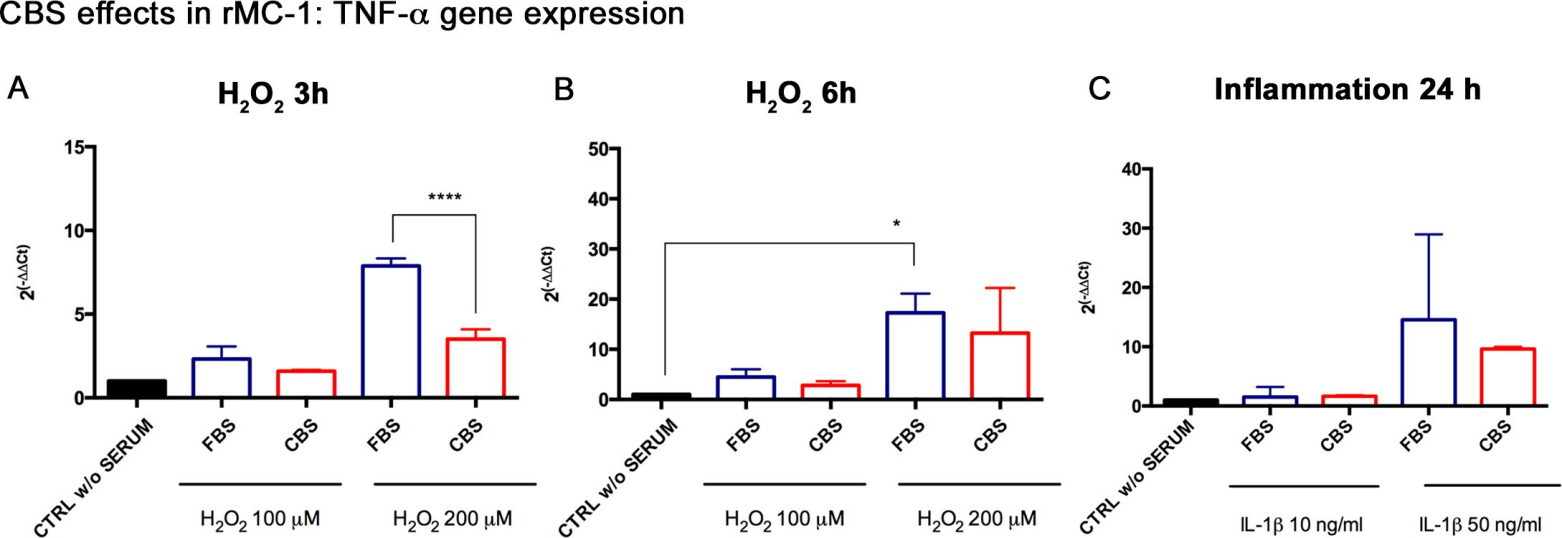
CBS reduces the expression of TNF-α in rMC-1 under stress conditions. The expression of TNF-α gene was analyzed by Real Time under oxidative stress for (A) 3 and (B) 6 h, and under (C) inflammation. Results are reported as fold changes relative to untreated controls, in DMEM without serum (CTRL w/o SERUM), and are expressed as mean ± standard deviation of three independent experiments. Statistical analysis was performed by ordinary one-way Anova, followed by Tukey’s multiple comparisons test. *, p<0.05; ****, p<0.0001.

In accordance with the improved cell viability observed under oxidative stress for 3 h, rMC-1 primed with CBS also displayed a reduction of GFAP mRNA, especially with 200 μM of H_2_O_2_ (Fig 2E). A significant down-regulation of GFAP transcription was also obtained under oxidative stress for 6 h with both H_2_O_2_ concentrations (Fig 2F). GFAP decrease was also reported in rMC-1 primed with CBS and exposed to inflammation with 50 ng/ml IL-1β for 24 h, as shown by Fig 2G. The expression of TNF-α was also analyzed, reporting an increase of its expression in a dose and time dependent fashion in rMC-1 grown with traditional FBS; conversely, the priming with CBS was associated with a lower expression of the inflammatory cytokine, both under oxidative stress (Fig 3A and 3B) and under inflammatory conditions (Fig 3C). These data supported the hypothesis that CBS reduces the toxic effects of microenvironment stress, attenuating the activation of Müller cells.

### CBS effects on human Müller cells: MIO-M1

**Fig 4:**
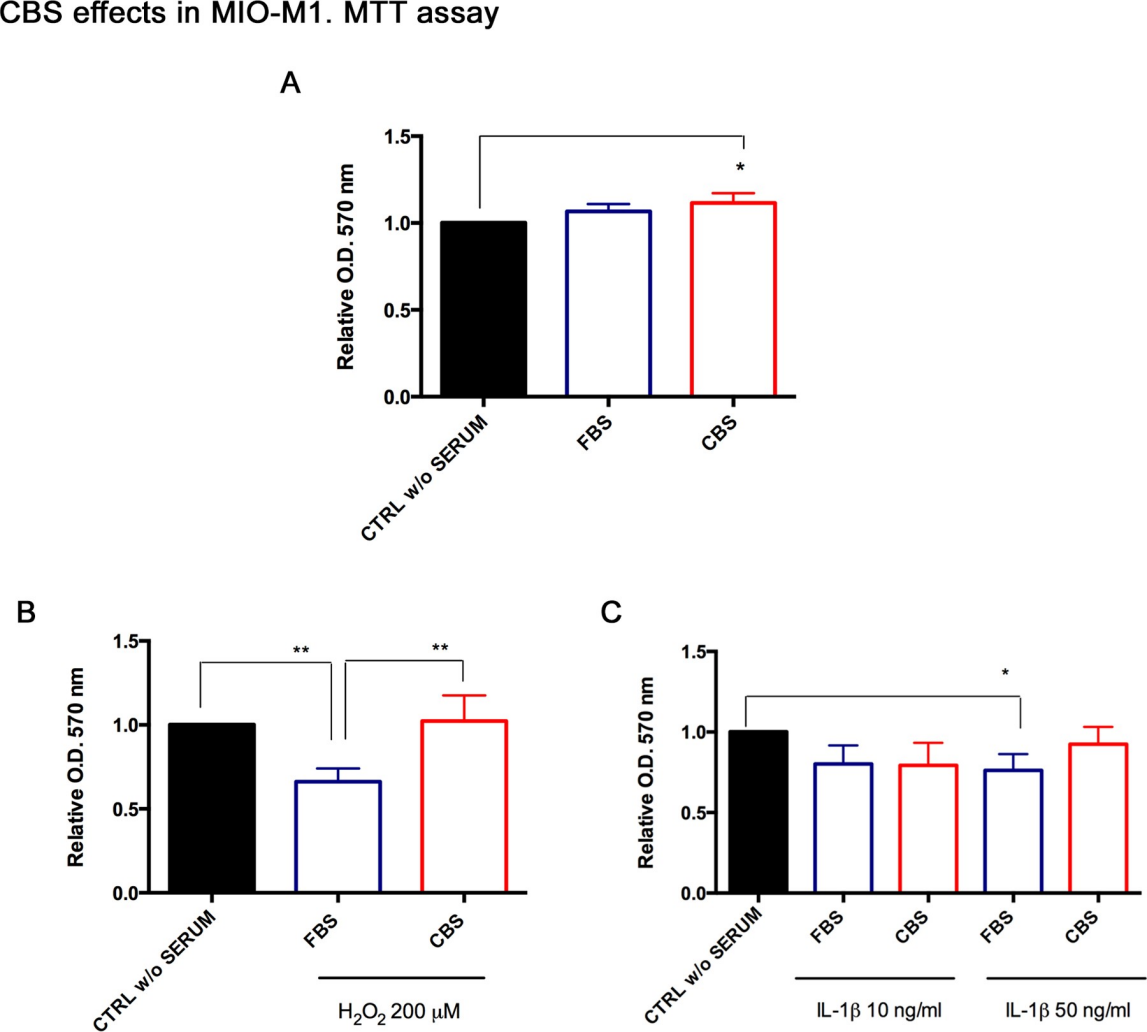
CBS improve MIO-M1 viability under stress conditions. MIO-M1 were grown with FBS or CBS for 24 h, and (A) cell viability was analyzed by MTT assay in order to evaluate possible effects of CBS on cell proliferation under control conditions. Then, MIO-M1 were exposed to (B) H_2_O_2_ 200 μM and (C) IL-1β (10–50 ng/ml) for 24 h, recording a significant increase of cell viability in comparison to FBS mainly under oxidative stress. MTT data were normalized to untreated controls, in DMEM without serum (CTRL w/o SERUM), as relative absorbance measured at an optical density (O.D.) of 570 nm, are reported as mean ± standard deviation of six independent experiments. Statistical analysis was performed by ordinary one-way Anova, followed by Tukey’s multiple comparisons test. *, p<0.05; **, p<0.01.

In parallel, we tested the effects of CBS on human model of Müller cells, MIO-M1. As described above, we selected a different treatment panel since rat and human cells displayed different behavior in terms of survival under stress conditions. The effects of CBS were measured through the MTT assay; preliminarily we explored whether CBS addition influences MIO-M1 viability and growth. As it can be observed in Fig 4A, there were no significant changes between FBS and CBS. Then, MIO-M1 grown with FBS or CBS were exposed to H_2_O_2_ (200 μM) and IL-1β (10–50 ng/ml) for 24 h. CBS resulted more effective at reducing cell death in presence of oxidative damage (Fig 4B). MIO-M1 survival was less affected, instead, by inflammatory induction (Fig 4C).

### Effects of CBS on GFAP expression in MIO-M1 under in vitro injury

**Fig 5:**
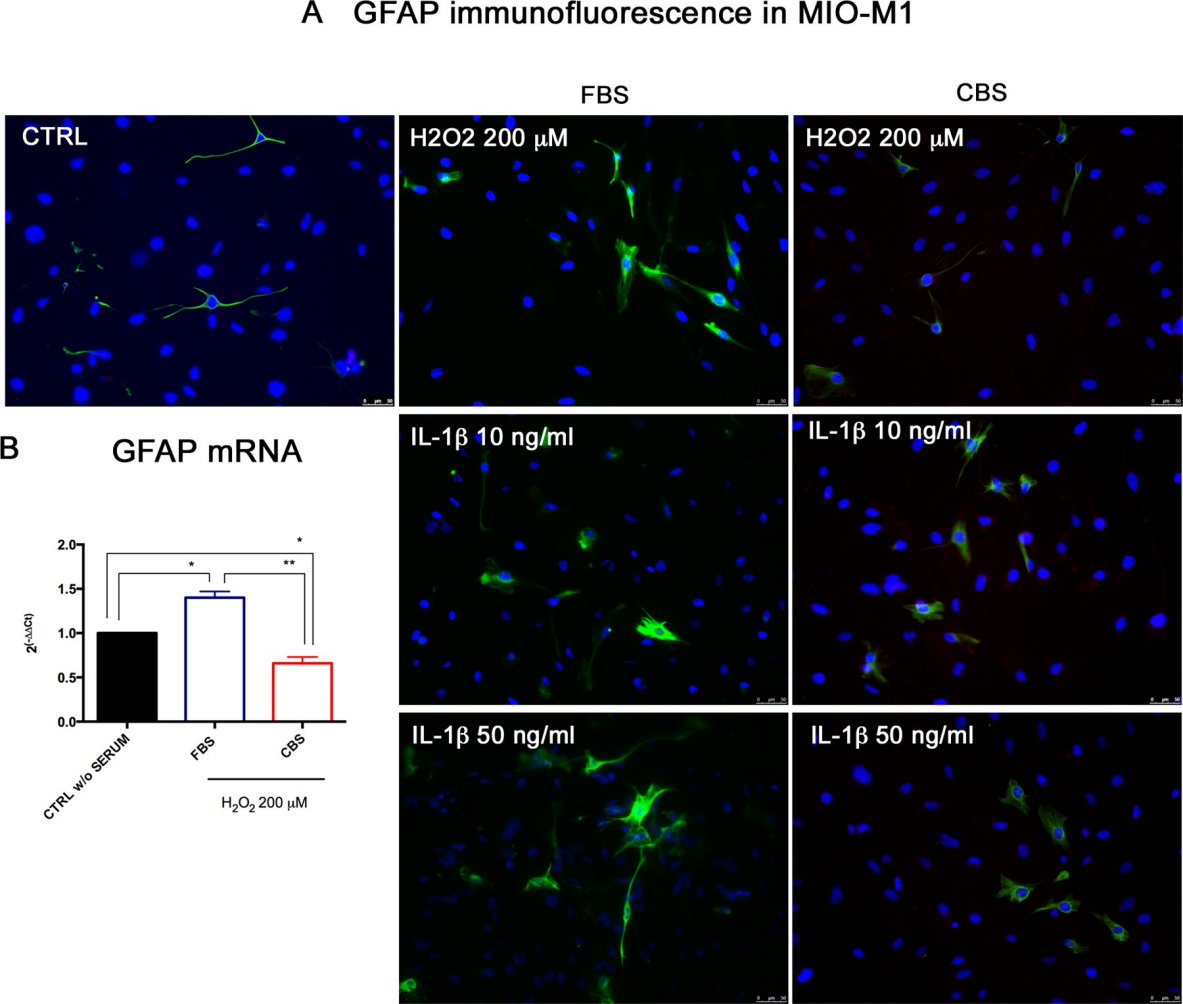
CBS reduces the expression of GFAP in MIO-M1 under stress conditions. MIO-M1 were grown with FBS or CBS for 24 h, exposed to oxidative and inflammatory injury and then analyzed in terms of GFAP expression, marker of gliosis. (A) Immunofluorescence reveals a pronounced expression of GFAP protein in MIO-M1 pre-grown with standard FBS (left panel), whereas a reduced positive number of cells are detectable in MIO-M1 cultured with CBS (right panel), especially in presence of IL-1β at 50 ng/ml. (B) Real Time PCR analysis of GFAP mRNA in presence of oxidative stress. Real Time data are reported as fold changes relative to untreated controls, in DMEM without serum, (CTRL w/o SERUM), and are expressed as mean ± standard deviation of three independent experiments. Statistical analysis was performed by ordinary one-way Anova, followed by Tukey’s multiple comparisons test. *, p<0.05; **, p<0.01.

In order to verify whether the beneficial role of CBS on MIO-M1 was mediated by a reduction of gliosis, we analyzed the expression of GFAP, marker of Müller cell activation. We observed a reduction of GFAP protein in MIO-M1 cultured with CBS, especially in presence of oxidative stress and inflammation with IL-1β 50 ng/ml (Fig 5A). Further a significant down-regulation of GFAP mRNA was recorded in MIO-M1 grown with CBS in comparison to serum free control cells and MIO-M1 cultured with standard FBS, under oxidative stress injury (Fig 5B). Conversely to protein, the expression of GFAP gene in MIO-M1 was not modulated under inflammatory stimuli, and did not manifest significant changes (S2 Fig).

### Effects of CBS on inflammatory mediators in MIO-M1 under in vitro injury

**Fig 6:**
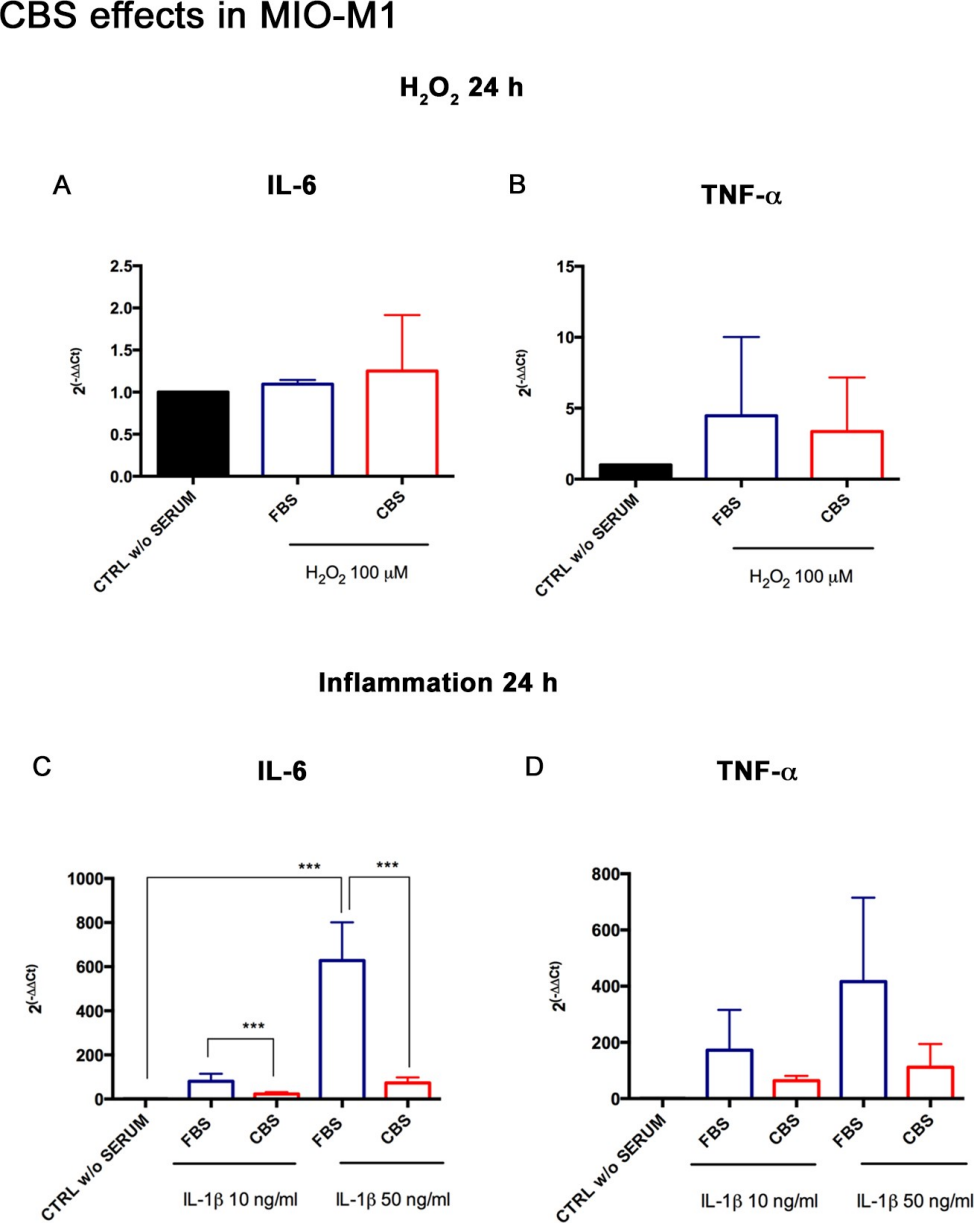
CBS reduces the expression of inflammatory genes in MIO-M1 under stress conditions. The analysis of the inflammatory genes IL-6 and TNF-α was performed in MIO-M1 grown with FBS or CBS, and then exposed to oxidative and inflammatory injury for 24 h. During oxidative stress, (A) IL-6 expression does not present significant variations and (B) TNF-α shows a decreasing trend with CBS. In presence of inflammatory stimulation, (C) IL-6 shows a significant down-regulation thanks to CBS pre-administration before damage, whereas (D) TNF-α resulted strongly reduced, even if not statistically significant. Real Time data are reported as fold changes relative to untreated controls, in DMEM without serum (CTRL w/o SERUM), and are expressed as mean ± standard deviation of three independent experiments. Statistical analysis was performed by ordinary one-way Anova, followed by Tukey’s multiple comparisons test. ***, p<0.001.

The inflammatory cascade is another index of Müller cell activation in response to damage inducers. We therefore analyzed the mRNA of inflammatory cytokine IL-6 and TNF-α in MIO-M1 primed with FBS or CBS and then exposed to the established treatment scheme. During oxidative stress, IL-6 and TNF-α did not undergo significant changes (Fig 6A and 6B). Interestingly, IL-6 displayed a significant down-regulation in MIO-M1 grown with CBS and exposed to IL-1β at both concentrations, in comparison to MIO-M1 grown with FBS (Fig 6C). TNF-α followed a decreasing trend.

### CBS effects in presence of TRK inhibitor

**Fig 7:**
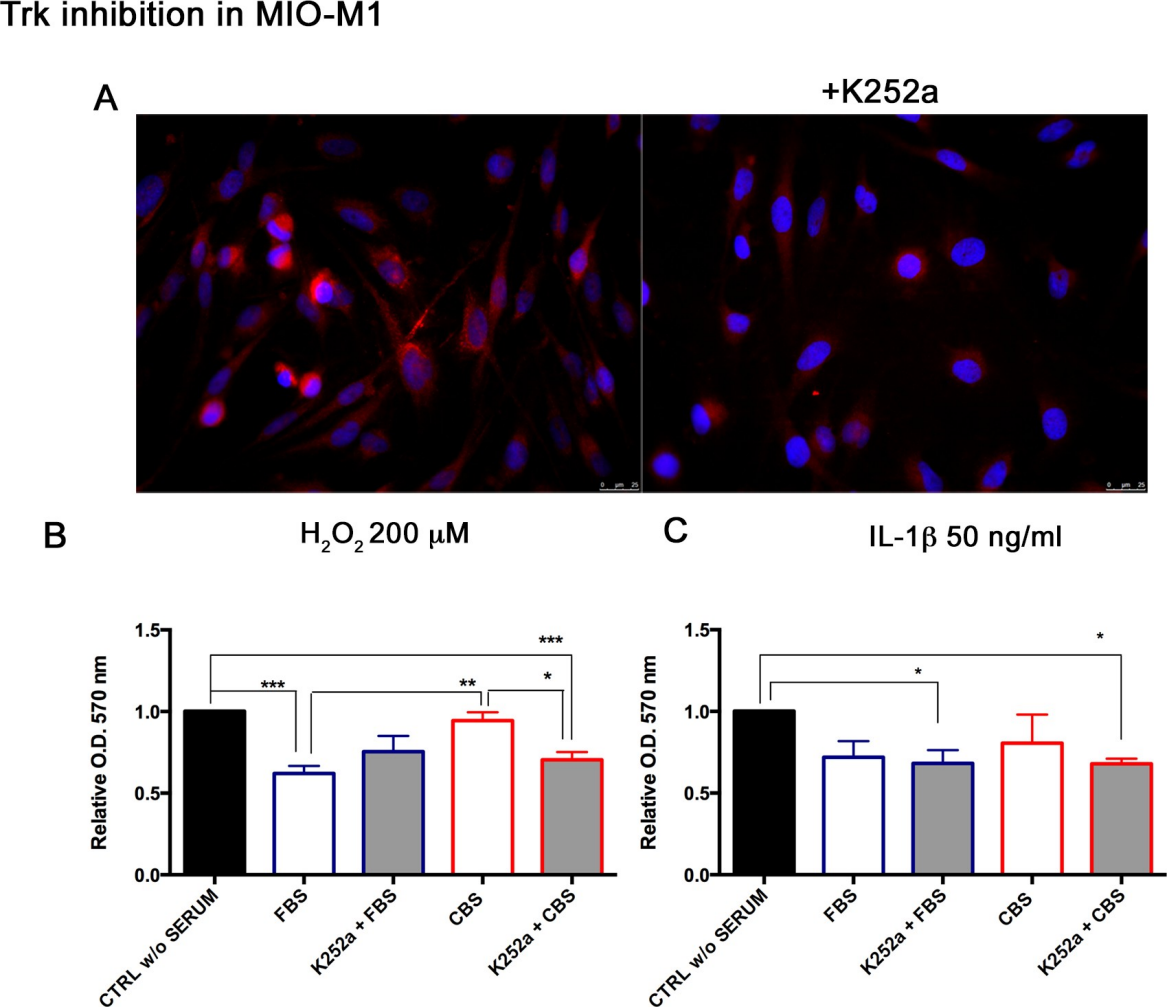
Trk inhibition reduces the CBS effects in MIO-M1. Before CBS addition to cell culture, MIO-M1 were treated with K252a in order to inhibit Trk receptor of neurotrophins. (A) Representative immunofluorescence of three independent experiments for detection of TrkA/B/C receptors after 1 h with K252a at 200 nm; red stain: TrkA/B/C; blue stain: nuclei. MTT assay was performed in MIO-M1 grown with CBS after Trk inhibition, under normal and injury conditions induced by (B) H_2_O_2_ 200 μM and (C) IL-1β (50 ng/ml) for 24 h. MTT data were normalized to untreated controls, in DMEM without serum (CTRL w/o SERUM), as relative absorbance measured at an optical density (O.D.) of 570 nm, are reported as mean ± standard deviation of three independent experiments. Statistical analysis was performed by ordinary one-way Anova, followed by Tukey’s multiple comparisons test. *, p<0.05; **, p<0.01; ***, p<0001.

**Fig 8:**
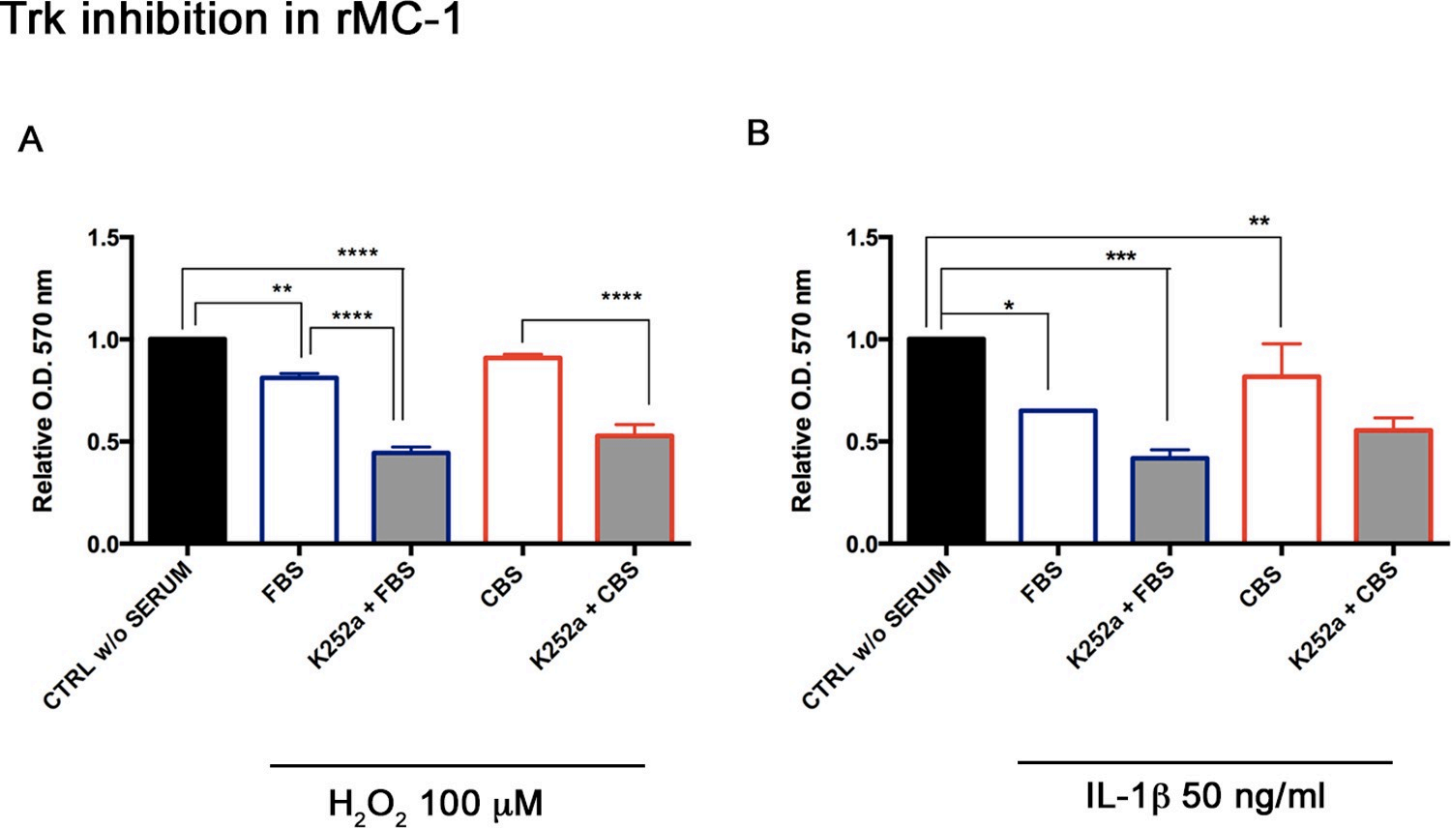
Trk inhibition reduces the CBS effects in rMC-1. Before CBS addition to cell culture, rMC-1 were treated with K252a in order to inhibit Trk receptor of neurotrophins. (A) As in MIO-M1, MTT assay was performed in rMC-1 grown with CBS after Trk inhibition, under normal and injury conditions induced by (A) H_2_O_2_ 100 μM and (C) IL-1β (50 ng/ml) for 24 h. MTT data were normalized to untreated controls, in DMEM without serum, (CTRL w/o SERUM), as relative absorbance measured at an optical density (O.D.) of 570 nm, are reported as mean ± standard deviation of three independent experiments. Statistical analysis was performed by ordinary one-way Anova, followed by Tukey’s multiple comparisons test. *, p<0.05; **, p<0.01.

Considering the high concentrations of neurotrophins (BDNF, NGF) in CBS, we asked whether the inhibition of the receptor Trk reduces the efficacy of CBS. Thus, we treated MIO-M1 with Trk inhibitor K252a (200 nM for 1 h) and confirmed its inhibition by immunofluorescence, where we observed a reduced expression of the receptor (Fig 7A). The quantification of Trk receptor expression was performed with ImageJ software, and measured as normalization of Trk positive areas to total area, confirming a 52% decrease of after K252a administration. Then, we repeated experiments in MIO-M1 for assessing CBS effects with/without K252a. The priming with CBS was associated with a recovery of cell viability, whereas the presence of K252a determined a significant reduction of CBS effect, especially under oxidative stress condition (mean values of absorbance in cells exposed to H_2_O_2_ damage were: 0.94 ± 0.05 in CBS-cells, 0.7 ±0.04 in CBS+K252a cells, p<0.05) (Fig 7B and 7C). This same experimental set was executed also in rMC-1, according to the following treatment conditions: H_2_O_2_ 100 μM and IL-1β 50 ng/ml for 24 h. MTT results reflected the same trend observed in MIO-M1. Indeed, the K252a administration reduced the effect of CBS on cell viability, corresponding to a lower cell survival under injury (mean values of absorbance in cells exposed to H_2_O_2_ damage were: 0.9 ± 0.3 in CBS cells, 0.5 ± 0.02 in CBS+K252a cells, p <0.05; mean values of absorbance in cells exposed to IL-1β stimulation were: 1.3 ± 0.4 in CBS cells, 0.5 ± 0.05 in CBS+K252a cells, p<0.01) (Fig 8A and 8B).

## Discussion

The present study was aimed at testing the in vitro effects of CBS in a model of retinal Müller glial cells, under stress conditions mimicking the neurodegenerative damage. The injury was determined in vitro by exposing cells to H_2_O_2_ and IL-1β, as inducers of oxidative stress and inflammation, respectively. The rationale to the administration of CBS to cell cultures derives from its high content of growth factors, including neurotrophins like BDNF, NGF and GDNF, essential to neuron survival and function. Retinal Müller cells have been selected as cell target for neuroprotection, since they perform pivotal roles for tissue physiology and healthy functioning. Among these, Müller cells secrete growth factors for neuron nourishment, possess an enzymatic metabolic system able to react at retinal injury and regulate angiogenesis and retinal blood flow [29]. Moreover, in some vertebrate models, Müller cells are able to dedifferentiate into retinal progenitors endowed with stemness features in response to injury stimuli, and transdifferentiate to cells with neuronal and photoreceptors characteristics [30,31]. However, the response to stress through gliosis can evolve into detrimental conditions, becoming markedly toxic for retinal itself. This condition is known as reactive gliosis, and it represents a hallmark of many neurodegenerative diseases. In addition to metabolic alterations, Müller cell impairment is also associated with the secretion of pro-inflammatory cytokines, like TNF-α and monocyte chemoattractant protein (MCP)-1 [32, 11]. Our data demonstrate that CBS is able to reduce the cell damage induced by oxidative stress and inflammatory cytokine, both on rat and human cell models of Müller cells. The improvement of cell viability and the lowering of gliosis markers GFAP suggest that CBS perform a potential protective function, by preserving cell death and its pathological activation under stress conditions. Thus, CBS can mitigate the gliosis, allowing Müller cells to react properly in presence of retinal injury and to exert a protective role on retinal ganglion cells. In addition, we also consider the effect of CBS on the expression of inflammatory cytokines in Müller cells in presence of stress damage. As shown by the analysis of IL-6 and TNF-α, a decreasing trend of their transcription is obtained in cells pre-cultured with CBS. This result is intriguing and implicates that the CBS supply to culture medium also meliorates the inflammatory activation, crucial mechanism responsible of the neurodegenerative diseases. These data can offer interesting cues for neuroprotection studies, supporting the results obtained both in animal model [14,15,17] and patients [33], where BDNF and NGF-based eye drops have been tested with beneficial effects on visual field.

The survival and protective functions of neurotrophins are mediated by the signaling cascade activated by the Trk receptor. The inhibition of Trk in hippocampal cells has been shown to reduce the beneficial effects of BDNF and NGF [34]. BDNF and NGF have been measured in CBS samples before administration to cell cultures, and our results show a high concentration of these growth factors. To this aim, we inhibited Trk by K252a, and then performed a novel set of experiments based on cell viability under stress conditions. We interestingly observed a significant reduction of cell viability in rMC-1 and MIO-M1 cultured with CBS and exposed to Trk inhibitor, suggesting that this step has impaired the effect of neurotrophins contained in CBS on cell survival in presence of injury. In addition, the viability rate of cells exposed to CBS with Trk inhibitor were comparable to cells grown with traditional FBS; these data consolidate the role of CBS in preventing cell damage and death under in vitro injury, and suggest that the neurotrophin signaling may represent a crucial neuroprotective mechanism in our model.

This study is a preliminary basis to further neuroprotection investigations for testing the impact of a natural mixture of neurotrophins contained in a blood product. Our data support the potential of CBS on an in vitro model, and the administration to a retinal Müller glial cell is novel and provides suggestions for exploring the application of these cells as target for therapeutic purposes.

## Supporting information

S1 Fig Levels of BDNF and NGF in CBS samples before and after heat inactivation. A slight reduction but without statistically significant changes was observed.
(TIF)

S2 Fig Analysis of GFAP mRNA in MIO-M1. Cells were primed with FBS or CBS and exposed to IL-1β (10–50 ng/ml). Real Time data are reported as fold changes relative to untreated controls, in DMEM without serum, and are expressed as mean ± standard deviation of three independent experiments.
(TIF)
