# Supplementary figures and images for: Effects of Cord Blood Serum (CBS) on viability of retinal Müller glial cells under in vitro injury
Source: PLoS One. 2020 Jun 4;15(6):e0234145. doi: 10.1371/journal.pone.0234145 (PMC7272066; doi:10.1371/journal.pone.0234145)

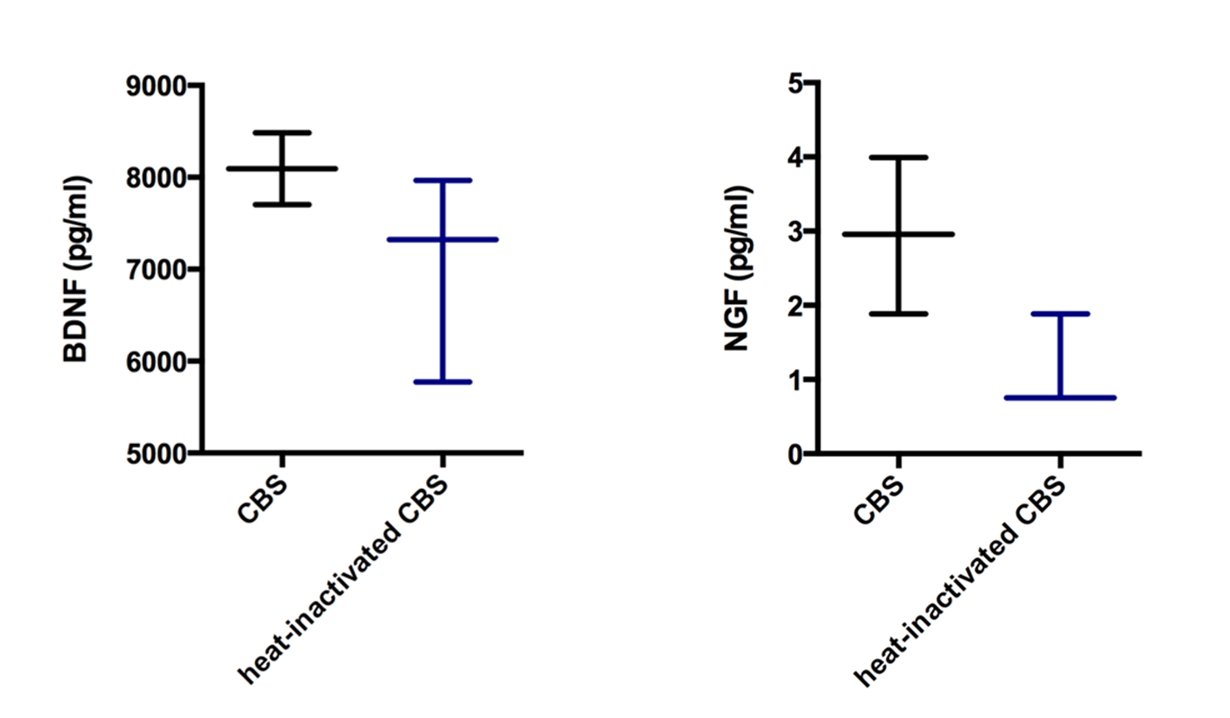

Supplement: S1 Fig — A slight reduction but without statistically significant changes was observed. (TIF) [file pone.0234145.s001.tif]

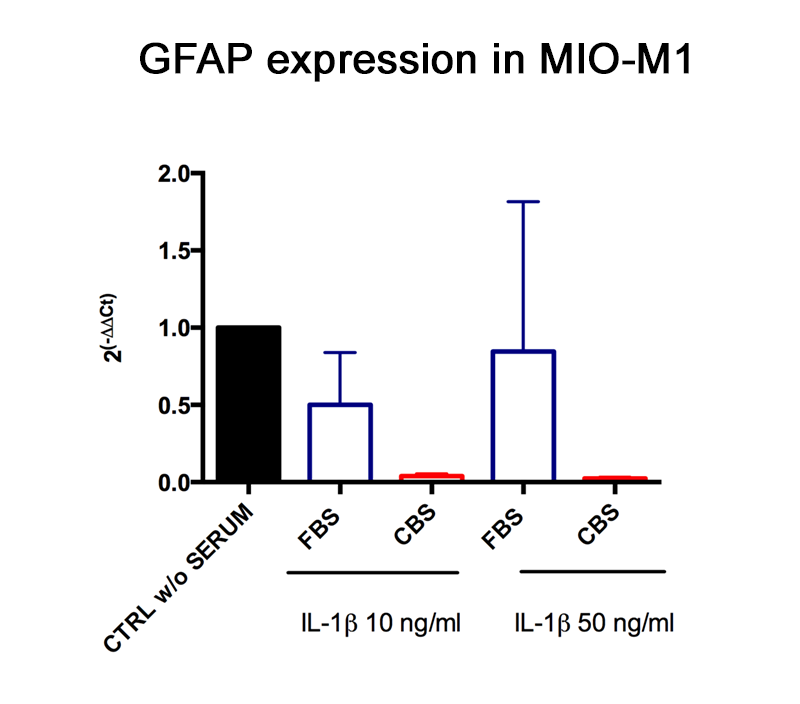

Supplement: S2 Fig — Cells were primed with FBS or CBS and exposed to IL-1β (10–50 ng/ml). Real Time data are reported as fold changes relative to untreated controls, in DMEM without serum, and are expressed as mean ± standard deviation of three independent experiments. (TIF) [file pone.0234145.s002.tif]
